# Special Issue: Thermo-Electric and Mechanical Properties of Carbon-Based Polymer

**DOI:** 10.3390/ma16196472

**Published:** 2023-09-29

**Authors:** Giovanni Spinelli, Vittorio Romano

**Affiliations:** 1 Faculty of Transport Sciences and Technologies, University of Benevento “Giustino Fortunato”, Via Raffaele Delcogliano 12, 82100 Benevento, Italy; 2 Department of Industrial Engineering, University of Salerno, Via Giovanni Paolo II, 132, 84084 Fisciano, Italy

Although on the one hand polymers are arousing increasing interest due to their remarkable properties in terms of lightness, cost-effectiveness, easy processing, and mechanical resistance, on the other hand, they still present several restrictions in practical applications [1,2]. These limitations are mainly due to their low electrical and thermal conductivity as well as their low resistance to chemical treatments, which might be concluded by weakening the overall mechanical strength. Consequently, polymers lose their functional ability; therefore, they need to be appropriately engineered to satisfy the growing demand for potential uses as adhesives, coatings in emerging technologies, in the field of microelectronics, as 3D printing feedstocks, and much more [3,4,5,6]. This Special Issue, entitled “Thermo-Electric and Mechanical Properties of Carbon-Based Polymer’’, aims to discuss the latest research findings in materials science and, in particular, in the field of novel polymer composites with improved mechanical, electrical, and thermal characteristics. One way to achieve this goal is to combine the polymer with highly conductive carbon-based fillers (such as carbon nanotubes, carbon nanofibers, and graphene and its derivates) to form advanced and multifunctional nanocomposites [7]. In fact, according to the percolation theory [8], as soon as a minimum filler concentration value is reached (the so-called percolation threshold) and exceeded, percolation paths are established within the polymer matrix, i.e., a continuous network of nanoparticles able to conduct an electric current to promote thermal transport and to confer greater mechanical strength to the resulting structures [9]. However, despite the results achieved so far [10,11], the observed properties for the nanocomposites are still far from those expected since their final overall properties are conditioned by a series of different factors not yet well understood, such as shape, aspect ratio, amount and functionalization of the fillers, interaction with the matrix and polarization at the interface, manufacturing process, temperature control, and so on. Consequently, further studies and research activities in the academic and industrial fields are required to fully benefit from all the potential offered by such novel materials. The research interests of this Special Issue include, but are not limited to, the design, the production, and the experimental characterization of nanocomposites; the investigation of new and environmentally friendly production methods; and the study of the structure–morphology relationship between the matrices and fillers to achieve multifunctional nanocomposites with advanced and customized properties. Moreover, computational studies and theoretical approaches are also highly appreciated in the current Special Issue, because they enable the discovery of new materials and understanding of their behaviors, to better investigate the existing materials and to design experiments [12,13,14,15].
